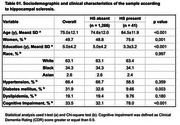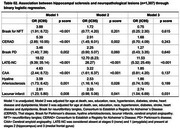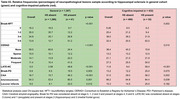# Bridging the Gap: Can Hippocampal Sclerosis Serve as a Surrogate Biomarker for LATE‐NC?

**DOI:** 10.1002/alz70862_109904

**Published:** 2025-12-23

**Authors:** Vitor Ribeiro Paes, Alberto Fernando Oliveira Justo, Caroline Matos Silva, Roberta Rodriguez, Renata Elaine Paraizo Leite, Claudia Kimie Suemoto, Carlos Augusto Pasqualucci, Eduardo Ferriolli, Wilson Jacob‐Filho, Lea T. Grinberg

**Affiliations:** ^1^ Physiopathology in Aging Laboratory (LIM‐22), University of Sao Paulo Medical School, São Paulo, São Paulo Brazil; ^2^ Physiopathology in Aging Laboratory (LIM‐22), University of São Paulo Medical School, São Paulo Brazil; ^3^ LIM44, Hospital das Clinicas HCFMUSP, Faculdade de Medicina, Universidade de Sao Paulo, Sao Paulo, Sao Paulo Brazil; ^4^ Biobank for Aging Studies of the University of São Paulo, São Paulo, São Paulo Brazil; ^5^ Division of Geriatrics, Department of Internal Medicine, University of São Paulo Medical School, São Paulo, São Paulo Brazil; ^6^ Faculdade de Medicina da Universidade de São Paulo, São Paulo Brazil; ^7^ University of Sao Paulo Medical School, São Paulo Brazil; ^8^ Memory and Aging Center, UCSF Weill Institute for Neurosciences, University of California, San Francisco, San Francisco, CA USA

## Abstract

**Background:**

Hippocampal sclerosis (HS) is characterized by neuronal loss and gliosis in the cornu Ammonis (CA) region of the hippocampus and is associated with epilepsy, hypoxia, and neurodegenerative diseases. In dementia, HS is increasingly recognized as a potential biomarker for Limbic‐predominant Age‐related TDP‐43 Encephalopathy neuropathological change (LATE‐NC). However, LATE‐NC currently lacks a validated in vivo biomarker. Since HS is detectable by MRI, a key question is whether HS could serve as a reliable proxy for underlying LATE‐NC pathology. This study examines the prevalence and pathological associations of HS across neurodegenerative diseases in a large population‐based brain bank to assess its potential as a biomarker for LATE‐NC.

**Method:**

Data were analyzed from the Biobank for Aging Study (BAS‐GEROLAB) in São Paulo, Brazil. Clinical and epidemiological information was obtained from next of kin using validated protocols, including the Clinical Dementia Rating (CDR) scale. Neuropathological assessments included morphological, vascular, and immunohistochemical analyses for beta‐ amyloid, tau, alpha‐synuclein, and TDP‐43. HS was defined as ≥70% neuronal loss in CA1 (unilateral). Individuals with a history of epilepsy were excluded.

**Result:**

Among 1,307 individuals, 36.9% were non‐White and 49.7% were women. HS was identified in 41 participants (3.1%), with higher prevalence among older individuals, women, and those with lower education levels (*p* < 0.001). HS was also significantly associated with hyaline arteriolosclerosis (*p* = 0.001) and diabetes mellitus (*p* = 0.003) (Table 1). Multivariate analysis confirmed associations between HS and LATE‐NC (*p* < 0.001) as well as lacunar infarcts (*p* = 0.031) (Table 2). The positive predictive value (PPV) of HS for TDP‐ 43 pathology was 48.8%, increasing to 59.4% among cognitively impaired individuals (*n* = 432) (Table 3). Among individuals with HS but no TDP‐43 pathology (*n* = 21), 48.4% had Braak‐AD stage ≥3, and 64.5% exhibited arteriolosclerosis.

**Conclusion:**

HS is relatively uncommon in this population‐based brain bank but is strongly associated with TDP‐43 pathology. However, vascular pathology also plays a significant role, particularly in populations with high cardiovascular risk. This limits its predictive value for LATE‐NC, even among cognitively impaired individuals. Given the absence of an in vivo biomarker for LATE‐NC, further research is needed to determine whether MRI‐detectable HS can serve as a reliable surrogate marker for LATE‐NC pathology, particularly in genetically diverse populations with multiple comorbidities.